# The use of digital tools to promote health in children: A systematic review of intervention studies: Giacomo Pietro Vigezzi

**DOI:** 10.1093/eurpub/ckac130.163

**Published:** 2022-10-25

**Authors:** G Dallagiacoma, P Ferrara, F Alberti, R Vecchio, GP Vigezzi, A Odone

**Affiliations:** 1 Department of Public Health, University of Pavia, Pavia, Italy; 2 Ca’ della Paglia College, Ghislieri Foundation, Pavia, Italy

## Abstract

**Background:**

Early childhood health interventions and educational programs are key to keeping children healthy and preventing disease during adulthood. Since several preventive strategies and campaigns targeting children have been proposed, the aim of this systematic review is to evaluate the effectiveness of digital-based interventions (e.g., cartoons, videos, video games, mobile apps, etc.) in promoting healthy behaviours in primary school-aged children.

**Methods:**

Following PRISMA guidelines, we searched three electronic databases (Medline, Embase, and Scopus) up to April 11, 2022. We included randomized and non-randomized experimental studies quantifying the effectiveness of digital or audio-visual-based health promotion interventions in childhood (up to 12 years of age).

**Results:**

The search strategy yielded a total of 1640 articles. Retrieved studies covered a wide range of health interventions - including a healthy diet, physical activity promotion, oral hygiene, skin cancer prevention, and different educational approaches (such as cartoons, interactive video games, etc.), mainly implemented in a school setting and comparing digital interventions to teacher-led interventions or no intervention at all. Data pooling suggests that digital and audio-visual-based health promotion interventions targeting children are effective in improving health literacy and healthy behaviours.

**Conclusions:**

This systematic review adds to the body of knowledge on health promotion in children and provides actionable measures to implement straightforward educational approaches in this specific population, empowering them to adopt preventive behaviours, and ultimately promoting health at the household and societal level.

**Key messages:**

- Health promotion has to be delivered during childhood and evidence is needed to identify effective interventions in this population.
- Digital and audio-visual tools have great potential to convey effective health promotion interventions in children and adults to be.

